# The impact of stress on body function: A review

**DOI:** 10.17179/excli2017-480

**Published:** 2017-07-21

**Authors:** Habib Yaribeygi, Yunes Panahi, Hedayat Sahraei, Thomas P. Johnston, Amirhossein Sahebkar

**Affiliations:** 1 Neurosciences Research Center, Baqiyatallah University of Medical Sciences, Tehran, Iran; 2 Clinical Pharmacy Department, Faculty of Pharmacy, Baqiyatallah University of Medical Sciences, Tehran, Iran; 3 Division of Pharmaceutical Sciences, School of Pharmacy, University of Missouri-Kansas City, Kansas City, Missouri, USA; 4 Biotechnology Research Center, Mashhad University of Medical Sciences, Mashhad, Iran

**Keywords:** stress, physiology, homeostasis

## Abstract

Any intrinsic or extrinsic stimulus that evokes a biological response is known as stress. The compensatory responses to these stresses are known as stress responses. Based on the type, timing and severity of the applied stimulus, stress can exert various actions on the body ranging from alterations in homeostasis to life-threatening effects and death. In many cases, the pathophysiological complications of disease arise from stress and the subjects exposed to stress, e.g. those that work or live in stressful environments, have a higher likelihood of many disorders. Stress can be either a triggering or aggravating factor for many diseases and pathological conditions. In this study, we have reviewed some of the major effects of stress on the primary physiological systems of humans.

## Abbreviations

ACTH: Adrenocorticotropic hormone

CNS: Central nervous system

CRH: Corticotropin releasing hormone

GI: Gastrointestinal

LTP: Long-term potentiation 

NMDA : N-methyl-D-aspartate

VTA: Ventral tegmental area 

## Stress and the Brain Function Complications

For a long time, researchers suggested that hormones have receptors just in the peripheral tissues and do not gain access to the central nervous system (CNS) (Lupien and Lepage, 2001[63]). However, observations have demonstrated the effect of anti-inflammatory drugs (which are considered synthetic hormones) on behavioral and cognitive disorders and the phenomenon called “Steroid psychosis” (Clark et al., 1952[16]). In the early sixties, neuropeptides were recognized as compounds devoid of effects on the peripheral endocrine system. However, it was determined that hormones are able to elicit biological effects on different parts of the CNS and play an important role in behavior and cognition (De Kloet, 2000[22]). In 1968, McEven suggested for the first time that the brain of rodents is capable of responding to glucocorticoid (as one of the operators in the stress cascade). This hypothesis that stress can cause functional changes in the CNS was then accepted (McEwen et al., 1968[74]). From that time on, two types of corticotropic receptors (glucocorticosteroids and mineralocorticoids) were recognized (de Kloet et al., 1999[23]). It was determined that the affinity of glucocorticosteroid receptors to cortisol and corticosterone was about one tenth of that of mineralocorticoids (de Kloet et al., 1999[23]). The hippocampus area has both types of receptors, while other points of the brain have only glucocorticosteroid receptors (de Kloet et al., 1999[23]). 

The effects of stress on the nervous system have been investigated for 50 years (Thierry et al., 1968[115]). Some studies have shown that stress has many effects on the human nervous system and can cause structural changes in different parts of the brain (Lupien et al., 2009[65]). Chronic stress can lead to atrophy of the brain mass and decrease its weight (Sarahian et al., 2014[100]). These structural changes bring about differences in the response to stress, cognition and memory (Lupien et al., 2009[65]). Of course, the amount and intensity of the changes are different according to the stress level and the duration of stress (Lupien et al., 2009[65]). However, it is now obvious that stress can cause structural changes in the brain with long-term effects on the nervous system (Reznikov et al., 2007[89]). Thus, it is highly essential to investigate the effects of stress on different aspects of the nervous system (Table 1(Tab. 1); References in Table 1: Lupien et al., 2001[63]; Woolley et al., 1990[122]; Sapolsky et al., 1990[99]; Gould et al., 1998[35]; Bremner, 1999[10]; Seeman et al., 1997[108]; Luine et al., 1994[62]; Li et al., 2008[60]; Scholey et al., 2014[101]; Borcel et al., 2008[9]; Lupien et al., 2002[66]).

## Stress and Memory

Memory is one of the important functional aspects of the CNS and it is categorized as sensory, short term, and long-term. Short term memory is dependent on the function of the frontal and parietal lobes, while long-term memory depends on the function of large areas of the brain (Wood et al., 2000[121]). However, total function of memory and the conversion of short term memory to long-term memory are dependent on the hippocampus; an area of the brain that has the highest density of glucocorticosteroid receptors and also represents the highest level of response to stress (Scoville and Milner, 1957[107]; Asalgoo et al., 2015[1]). Therefore, during the past several decades, the relationship between the hippocampus and stress have been hotly debated (Asalgoo et al., 2015[1]; Lupien and Lepage, 2001[63]). In 1968, it was proven that there were cortisol receptors in the hippocampus of rats (McEwen et al., 1968[74]). Later, in 1982, by using specific agonists of glucocorticosteroid and mineralocorticoid receptors, the existence of these two receptors in the brain and hippocampus area of rats was proven (Veldhuis et al., 1982[119]). It should also be noted that the amygdala is very important to assessing the emotional experiences of memory (Roozendaal et al., 2009[91]). 

The results of past studies have demonstrated the effect of stress on the process of memory (Ghodrat et al., 2014[32]). Various studies have shown that stress can cause functional and structural changes in the hippocampus section of the brain (McEwen, 1999[72]). These structural changes include atrophy and neurogenesis disorders (Lupien and Lepage, 2001[63]). Also, chronic stress and, consequently, an increase in plasma cortisol, leads to a reduction in the number of dendritic branches (Woolley et al., 1990[122]) and the number of neurons (Sapolsky et al., 1990[99]), as well as structural changes in synaptic terminals (Sapolsky et al., 1990[99]) and decreased neurogenesis in the hippocampus tissue (Gould et al., 1998[35]). Glucocorticosteroids can induce these changes by either effecting the cellular metabolism of neurons (Lawrence and Sapolsky, 1994[58]), or increasing the sensitivity of hippocampus cells to stimulatory amino acids (Sapolsky and Pulsinelli, 1985[98]) and/or increasing the level of extracellular glutamate (Sapolsky and Pulsinelli, 1985[98]).

High concentrations of stress hormones can cause declarative memory disorders (Lupien and Lepage, 2001[63]). Animal studies have shown that stress can cause a reversible reduction in spatial memory as a result of atrophy of the hippocampus (Luine et al., 1994[62]). In fact, high plasma concentrations of glucocorticosteroids for extended periods of time can cause atrophy of the hippocampus leading to memory disorders (Issa et al., 1990[45]). Additionally, people with either Cushing's syndrome (with an increased secretion of glucocorticosteroids), or people who receive high dosages of exogenous synthetic anti-inflammatory drugs, are observed to have atrophy of the hippocampus and associated memory disorders (Ling et al., 1981[61]). MRI images taken from the brains of people with post-traumatic stress disorder (PTSD) have demonstrated a reduction in the volume of the hippocampus along with neurophysiologic effects such as a weak verbal memory (Bremner, 1999[10]). Several human studies have suggested that even common therapeutic doses of glucocorticosteroids and dexamethasone can cause problems with explicit memory (Keenan et al., 1995[49]; Kirschbaum et al., 1996[53]). Thus, there is an inverse relationship between the level of cortisol and memory (Ling et al., 1981[61]), such that increasing levels of plasma cortisol following prolonged stress leads to a reduction in memory (Kirschbaum et al., 1996[53]), which improves when the level of plasma cortisol decreases (Seeman et al., 1997[108]). 

Stress also has negative effects on learning. Results from hippocampus-dependent loading data demonstrate that subjects are not as familiar with a new environment after having been exposed to a new environment (Bremner, 1999[10]). Moreover, adrenal steroids lead to alteration in long-term potentiation (LTP), which is an important process in memory formation (Bliss and Lømo, 1973[7]). 

Two factors are involved in the memory process during stress. The first is noradrenaline, which creates emotional aspects of memories in the basolateral amygdala area (Joëls et al., 2011[47]). Secondly, this process is facilitated by corticosteroids. However, if the release of corticosteroids occurs a few hours earlier, it causes inhibition of the amygdala and corresponding behaviors (Joëls et al., 2011[47]). Thus, there is a mutual balance between these two hormones for creating a response in the memory process (Joëls et al., 2011[47]). 

Stress does not always affect memory. Sometimes, under special conditions, stress can actually improve memory (McEwen and Lupien, 2002[71]). These conditions include non-familiarity, non-predictability, and life-threatening aspects of imposed stimulation. Under these specific conditions, stress can temporarily improve the function of the brain and, therefore, memory. In fact, it has been suggested that stress can sharpen memory in some situations (Schwabe et al., 2010[105]). For example, it has been shown that having to take a written examination can improve memory for a short period of time in examination participants. Interestingly, this condition is associated with a decrease in the level of cortisol in the saliva (Vedhara et al., 2000[118]). Other studies have shown that impending stress before learning occurs can also lead to either an increase in the power of memory (Domes et al., 2002[27]; Schwabe et al., 2008[102]), or decrease in the capacity for memory (Diamond et al., 2006[26]; Kirschbaum et al., 1996[53]). This paradox results from the type of imposed stress and either the degree of emotional connection to the stressful event (Payne et al., 2007[83]; Diamond et al., 2007[25]), or the period of time between the imposing stress and the process of learning (Diamond et al., 2007[25]). 

The process of strengthening memory is usually reinforced after stress (Schwabe et al., 2012[103]). Various studies on animal and human models have shown that administration of either glucocorticosteroids, or stress shortly after learning has occurred facilitates memory (Schwabe et al., 2012[103]). Also, it has been shown that glucocorticosteroids (not mineralocorticoids) are necessary to improve learning and memory (Lupien et al., 2002[66]). However, the retrieval of events in memory after exposure to stress will be decreased (Schwabe et al., 2012[103]), which may result from the competition of updated data for storage in memory in a stressful state (de Kloet et al., 1999[23]). Some investigations have shown that either exposure to stress, or injection of glucocorticosteroids before a test to assess retention, decreases the power of memory in humans and rodents (Schwabe and Wolf, 2009[104]).

In summary, it has been concluded that the effect of stress on memory is highly dependent on the time of exposure to the stressful stimulus and, in terms of the timing of the imposed stress, memory can be either better or worse (Schwabe et al., 2012[103]). Moreover, recent studies have shown that using a specific-timed schedule of exposure to stress not only affects hippocampus-dependent memory, but also striatum-dependent memory, which highlights the role of timing of the imposed stressful stimulus (Schwabe et al., 2010[105]).

## Stress, Cognition and Learning

Cognition is another important feature of brain function. Cognition means reception and perception of perceived stimuli and its interpretation, which includes learning, decision making, attention, and judgment (Sandi, 2013[95]). Stress has many effects on cognition that depend on its intensity, duration, origin, and magnitude (Sandi, 2013[95]). Similar to memory, cognition is mainly formed in the hippocampus, amygdala, and temporal lobe (McEwen and Sapolsky, 1995[73]). The net effect of stress on cognition is a reduction in cognition and thus, it is said that any behavioral steps undertaken to reduce stress leads to increase in cognition (Scholey et al., 2014[101]). In fact, stress activates some physiological systems, such as the autonomic nervous system, central neurotransmitter and neuropeptide system, and the hypothalamus-pituitary-adrenal axis, which have direct effects on neural circuits in the brain involved with data processing (Sandi, 2013[95]). Activation of stress results in the production and release of glucocorticosteroids. Because of the lipophilic properties of glucocorticosteroids, they can diffuse through the blood-brain barrier and exert long-term effects on processing and cognition (Sandi, 2013[95]).

It appears that being exposed to stress can cause pathophysiologic changes in the brain, and these changes can be manifested as behavioral, cognitive, and mood disorders (Li et al., 2008[60]). In fact, studies have shown that chronic stress can cause complications such as increased IL-6 and plasma cortisol, but decreased amounts of cAMP responsive element binding protein and brain-derived neurotrophic factor (BDNF), which is very similar to what is observed in people with depression and mood disorders that exhibit a wide range of cognitive problems (Song et al., 2006[114]). Additionally, the increased concentrations of inflammatory factors, like interleukins and TNF-α (which play an important role in creating cognitive disorders), proves a physiologic relationship between stress and mood-based cognitive disorders (Solerte et al., 2000[113]; Marsland et al., 2006[68]; Li et al., 2008[60]). Studies on animals suggest that cognitive disorders resulting from stress are created due to neuroendocrine and neuroamine factors and neurodegenerative processes (Li et al., 2008[60]). However, it should be noted that depression may not always be due to the over activation of the physiological-based stress response (Osanloo et al., 2016[81]).

Cognitive disorders following exposure to stress have been reported in past studies (Lupien and McEwen, 1997[64]). Stress has effects on cognition both acutely (through catecholamines) and chronically (through glucocorticosteroids) (McEwen and Sapolsky, 1995[73]). Acute effects are mainly caused by beta-adrenergic effects, while chronic effects are induced in a long-term manner by changes in gene expression mediated by steroids (McEwen and Sapolsky, 1995[73]). In general, many mechanisms modulate the effects of stress on cognition (McEwen and Sapolsky, 1995[73]; Mendl, 1999[75]). For instance, adrenal steroids affect the function of the hippocampus during cognition and memory retrieval in a biphasic manner (McEwen and Sapolsky, 1995[73]). In chronic stress, these steroids can destroy neurons with other stimulatory neurotransmitters (Sandi, 2013[95]). Exposure to stress can also cause disorders in hippocampus-related cognition; specifically, spatial memory (Borcel et al., 2008[9]; Sandi et al., 2003[96]). Additionally, stress can halt or decrease the genesis of neurons in the dentate gyrus area of the hippocampus (this area is one of the limited brain areas in which neurogenesis occurs in adults) (Gould and Tanapat, 1999[34]; Köhler et al., 2010[54]). Although age is a factor known to affect cognition, studies on animals have demonstrated that young rats exposed to high doses of adrenal steroids show the same level of decline in their cognition as older adult animals with normal plasma concentrations of glucocorticoids (Landfield et al., 1978[57]). Also, a decrease in the secretion of glucocorticosteroids causes preservation of spatial memory in adults and has also been shown to have neuroprotective effects (Montaron et al., 2006[78]). Other studies have shown that stress (or the injection of adrenal steroids) results in varied effects on cognition. For instance, injection of hydrocortisone at the time of its maximum plasma concentration (in the afternoon) leads to a decrease in reaction time and improves cognition and memory (Lupien et al., 2002[66]). 

In summary, the adverse effects of stress on cognition are diverse and depend on the type, timing, intensity, and duration (Sandi, 2013[95]). Generally, it is believed that mild stress facilitates an improvement in cognitive function, especially in the case of virtual or verbal memory. However, if the intensity of stress passes beyond a predetermined threshold (which is different in each individual), it causes cognitive disorders, especially in memory and judgment. The disruption to memory and judgment is due to the effects of stress on the hippocampus and prefrontal cortex (Sandi, 2013[95]). Of course, it must be realized that factors like age and gender may also play a role in some cognitive disorders (Sandi, 2013[95]). Importantly, it should be emphasized that different people may exhibit varied responses in cognition when exposed to the very same stressful stimulus (Hatef et al., 2015[39]).

## Stress and Immune System Functions

The relationship between stress and the immune system has been considered for decades (Khansari et al., 1990[50]; Dantzer and Kelley, 1989[21]). The prevailing attitude between the association of stress and immune system response has been that people under stress are more likely to have an impaired immune system and, as a result, suffer from more frequent illness (Khansari et al., 1990[50]). Also, old anecdotes describing resistance of some people to severe disease using the power of the mind and their thought processes, has promoted this attitude (Khansari et al., 1990[50]). In about 200 AC, Aelius Galenus (Galen of Pergamon) declared that melancholic women (who have high levels of stress and, thus, impaired immune function) are more likely to have cancer than women who were more positive and exposed to less stress (Reiche et al., 2004[88]). This may be the first recorded case about the relationship between the immune system and stress. In an old study in the early 1920's, researchers found that the activity of phagocytes in tuberculosis decreased when emotional stress was induced. In fact, it was also suggested that living with stress increases the risk of tuberculosis by suppressing the immune system (Ishigami, 1919[44]). Following this study, other researchers suggested that the probability of disease appearance increases following a sudden, major, and extremely stressful life style change (Holmes and Rahe, 1967[41]; Calabrese et al., 1987[12]). 

Over the past several decades, there have been many studies investigating the role of stress on immune system function (Dantzer and Kelley, 1989[21]; Segerstrom and Miller, 2004[109]). These studies have shown that stress mediators can pass through the blood-brain barrier and exert their effects on the immune system (Khansari et al., 1990[50]). Thus, the effect of stress on the immune system is now an accepted relationship or association.

Stress can affect the function of the immune system by modulating processes in the CNS and neuroendocrine system (Khansari et al., 1990[50]; Kiecolt-Glaser and Glaser, 1991[51]). Following stress, some neuroendocrine and neural responses result in the release of corticotropin-releasing hormone (CRH), adrenocorticotropic hormone (ACTH), and other stress mediators (Carrasco and Van de Kar, 2003[13]). However, evidence suggests that the lymphatic system, which is a part of the immune system, also plays a role in releasing these mediators (Khansari et al., 1990[50]). For instance, thymus peptides, such as thymopentine, thymopoietin, and thymosin fraction-5, cause an increase in ACTH production (Goya et al., 1993[36]). Additionally, the existence of CRH in thymus has been proven (Redei, 1992[87]). It has also been proven that interleukin-1 released from phagocytes has a role in ACTH secretion (Berkenbosch et al., 1987[4]). On the other hand, natural or synthetic glucocorticosteroids (which are the final stress operators) are known as anti-inflammatory drugs and immune suppressants and their role in the inhibition of lymphocytes and macrophages has been demonstrated as well (Elenkov et al., 1999[28]; Reiche et al., 2004[88]). Moreover, their role in inhibiting the production of cytokines and other immune mediators and decreasing their effect on target cells during exposure to stress has also been determined (Reiche et al., 2004[88]).

In addition to adrenal steroids, other hormones are affected during stress. For example, the secretion of growth hormone will be halted during severe stress. A study showed that long-term administration of CRH into the brain ventricles leads to a cessation in the release of growth hormone (Rivier and Vale, 1985[90]). Stress also causes the release of opioid peptides to be changed during the time period over which the person is exposed to stress (McCarthy et al., 2001[70]). In fact, stress modifies the secretion of hormones that play a critical role in the function of the immune system (Khansari et al., 1990[50]). To date, it has been shown that various receptors for a variety of hormones involved in immune system function are adversely affected by stress. For example, ACTH, vasoactive intestinal peptide (VIP), substance P, growth hormone, prolactin, and steroids all have receptors in various tissues of the immune system and can modulate its function (De la Fuente et al., 1996[24]; Gala, 1991[30]; Mantyh, 1991[67]). In addition, active immune cells are also able to secrete several hormones; thus, some researchers believe that these hormones, as mediators of immune system, play a significant role in balancing its function (Blalock et al., 1985[6]).

Severe stress can lead to malignancy by suppressing the immune system (Reiche et al., 2004[88]). In fact, stress can decrease the activity of cytotoxic T lymphocytes and natural killer cells and lead to growth of malignant cells, genetic instability, and tumor expansion (Reiche et al., 2004[88]). Studies have shown that the plasma concentration of norepinephrine, which increases after the induction stress, has an inverse relationship with the immune function of phagocytes and lymphocytes (Reiche et al., 2004[88]). Lastly, catecholamines and opioids that are released following stress have immune-suppressing properties (Reiche et al., 2004[88]). 

## Stress and the Function of the Cardiovascular System

The existence of a positive association between stress and cardiovascular disease has been verified (Rozanski et al., 1999[93]). Stress, whether acute or chronic, has a deleterious effect on the function of the cardiovascular system (Rozanski et al., 1999[93]; Kario et al., 2003[48]; Herd, 1991[40]). The effects of stress on the cardiovascular system are not only stimulatory, but also inhibitory in nature (Engler and Engler, 1995[29]). It can be postulated that stress causes autonomic nervous system activation and indirectly affects the function of the cardiovascular system (Lazarus et al., 1963[59]; Vrijkotte et al., 2000[120]). If these effects occur upon activation of the sympathetic nervous system, then it mainly results in an increase in heart rate, strength of contraction, vasodilation in the arteries of skeletal muscles, a narrowing of the veins, contraction of the arteries in the spleen and kidneys, and decreased sodium excretion by the kidneys (Herd, 1991[40]). Sometimes, stress activates the parasympathetic nervous system (Pagani et al., 1991[82]). Specifically, if it leads to stimulation of the limbic system, it results in a decrease, or even a total stopping of the heart-beat, decreased contractility, reduction in the guidance of impulses by the heart stimulus-transmission network, peripheral vasodilatation, and a decline in blood pressure (Cohen et al., 2000[17]). Finally, stress can modulate vascular endothelial cell function and increase the risk of thrombosis and ischemia, as well as increase platelet aggregation (Rozanski et al., 1999[93]).

The initial effect of stress on heart function is usually on the heart rate (Vrijkotte et al., 2000[120]). Depending upon the direction of the shift in the sympatho-vagal response, the heart beat will either increase or decrease (Hall et al., 2004[38]). The next significant effect of stress on cardiovascular function is blood pressure (Laitinen et al., 1999[56]). Stress can stimulate the autonomic sympathetic nervous system to increase vasoconstriction, which can mediate an increase in blood pressure, an increase in blood lipids, disorders in blood clotting, vascular changes, atherogenesis; all, of which, can cause cardiac arrhythmias and subsequent myocardial infarction (Rozanski et al., 1999[93]; Vrijkotte et al., 2000[120]; Sgoifo et al., 1998[111]). These effects from stress are observed clinically with atherosclerosis and leads to an increase in coronary vasoconstriction (Rozanski et al., 1999[93]). Of course, there are individual differences in terms of the level of autonomic-based responses due to stress, which depends on the personal characteristics of a given individual (Rozanski et al., 1999[93]). Thus, training programs for stress management are aimed at reducing the consequences of stress and death resulting from heart disease (Engler and Engler, 1995[29]). In addition, there are gender-dependent differences in the cardiovascular response to stress and, accordingly, it has been estimated that women begin to exhibit heart disease ten years later that men, which has been attributed to the protective effects of the estrogen hormone (Rozanski et al., 1999[93]).

Studies have shown that psychological stress can cause alpha-adrenergic stimulation and, consequently, increase heart rate and oxygen demand (Rozanski et al., 1998[92], 1999[93]; Jiang et al., 1996[46]). As a result, coronary vasoconstriction is enhanced, which may increase the risk of myocardial infarction (Yeung et al., 1991[124]; Boltwood et al., 1993[8]; Dakak et al., 1995[20]). Several studies have demonstrated that psychological stress decreases the microcirculation in the coronary arteries by an endothelium-dependent mechanism and increases the risk of myocardial infarction (Dakak et al., 1995[20]). On the other hand, mental stress indirectly leads to potential engagement in risky behaviors for the heart, such as smoking, and directly leads to stimulation of the neuroendocrine system as part of the autonomic nervous system (Hornstein, 2004[43]). It has been suggested that severe mental stress can result in sudden death (Pignalberi et al., 2002[84]). Generally, stress-mediated risky behaviors that impact cardiovascular health can be summarized into five categories: an increase in the stimulation of the sympathetic nervous system, initiation and progression of myocardial ischemia, development of cardiac arrhythmias, stimulation of platelet aggregation, and endothelial dysfunction (Wu, 2001[123]).

## Stress and Gastrointestinal Complications

The effects of stress on nutrition and the gastrointestinal (GI) system can be summarized with two aspects of GI function.

First, stress can affect appetite (Bagheri Nikoo et al., 2014[2]; Halataei et al., 2011[37]; Ranjbaran et al., 2013[86]). This effect is related to involvement of either the ventral tegmental area (VTA), or the amygdala *via* N-methyl-D-aspartate (NMDA) glutamate receptors (Nasihatkon et al., 2014[80]; Sadeghi et al., 2015[94]). However, it should also be noted that nutrition patterns have effects on the response to stress (Ghanbari et al., 2015[31]), and this suggests a bilateral interaction between nutrition and stress. 

Second, stress adversely affects the normal function of GI tract. There are many studies concerning the effect of stress on the function of the GI system (Söderholm and Perdue, 2001[112]; Collins, 2001[18]). For instance, studies have shown that stress affects the absorption process, intestinal permeability, mucus and stomach acid secretion, function of ion channels, and GI inflammation (Collins, 2001[18]; Nabavizadeh et al., 2011[79]). Stress also increases the response of the GI system to inflammation and may reactivate previous inflammation and accelerate the inflammation process by secretion of mediators such as substance P (Collins, 2001[18]). As a result, there is an increase in the permeability of cells and recruitment of T lymphocytes. Lymphocyte aggregation leads to the production of inflammatory markers, activates key pathways in the hypothalamus, and results in negative feedback due to CRH secretion, which ultimately results in the appearance of GI inflammatory diseases (Collins, 2001[18]). This process can reactivate previous silent colitis (Million et al., 1999[76]; Qiu et al., 1999[85]). Mast cells play a crucial role in stress-induced effects on the GI system, because they cause neurotransmitters and other chemical factors to be released that affect the function of the GI system (Konturek et al., 2011[55]).

Stress can also alter the functional physiology of the intestine (Kiliaan et al., 1998[52]). Many inflammatory diseases, such as Crohn's disease and other ulcerative-based diseases of the GI tract, are associated with stress (Hommes et al., 2002[42]). It has been suggested that even childhood stress can lead to these diseases in adulthood (Schwartz and Schwartz, 1983[106]). Irritable bowel syndrome, which is a disease with an inflammatory origin, is highly related to stress (Gonsalkorale et al., 2003[33]). Studies on various animals suggest the existence of inflammatory GI diseases following induction of severe stress (Qiu et al., 1999[85]; Collins et al., 1996[19]). Additionally, pharmacological interventions, in an attempt to decrease the response of CRH to stress, have been shown to result in an increase in GI diseases in rats (Million et al., 1999[76]).

Altering the permeability of the mucosal membrane by perturbing the functions of mucosal mast cells may be another way that stress causes its effects on the GI system, since this is a normal process by which harmful and toxic substances are removed from the intestinal lumen (Söderholm and Perdue, 2001[112]). Also, stress can both decrease the removal of water from the lumen, as well as induce sodium and chloride secretion into the lumen. This most likely occurs by increasing the activity of the parasympathetic nervous system (Barclay and Turnberg, 1987[3]). Moreover, physical stress, such as trauma or surgery, can increase luminal permeability (Söderholm and Perdue, 2001[112]) (Table 2(Tab. 2); References in Table 2: Halataei et al., 2011[37]; Ranjbaran et al., 2013[86]; Mönnikes et al., 2001[77]; Collins, 2001[18]; Nabavizadeh et al., 2011[79]; Barclay and Turnberg, 1987[3]; Million et al., 1999[76]; Gonsalkorale et al., 2003[33]). 

Stress also affects movement of the GI tract. In this way, it prevents stomach emptying and accelerates colonic motility (Mönnikes et al., 2001[77]). In the case of irritable bowel syndrome, stress increases the movement (contractility and motility) of the large intestine (Mönnikes et al., 2001[77]). Previous studies have revealed that CRH increases movement in the terminal sections of the GI tract and decreases the movements in the proximal sections of the GI tract (Mönnikes et al., 2001[77]). A delay in stomach emptying is likely accomplished through CRH-2 receptors, while type 1 receptors affect the colon (Mönnikes et al., 2001[77]). The effects produced by CRH are so prominent that CRH is now considered an ideal candidate for the treatment of irritable bowel syndrome (Martinez and Taché, 2006[69]). When serotonin is released in response to stress (Chaouloff, 2000[14]), it leads to an increase in the motility of the colon by stimulating 5HT-3 receptors (Mönnikes et al., 2001[77]). Moreover, it has also been suggested that stress, especially mental and emotional types of stress, increase visceral sensitivity and activate mucosal mast cells (Mönnikes et al., 2001[77]). Stimulation of the CNS by stress has a direct effect on GI-specific nervous system (*i.e.*, the myenteric system or plexus) and causes the above mentioned changes in the movements of the GI tract (Bhatia and Tandon, 2005[5]). In fact, stress has a direct effect on the brain-bowel axis (Konturek et al., 2011[55]). Various clinical studies have suggested a direct effect of stress on irritable bowel syndrome, intestinal inflammation, and peptic ulcers (Konturek et al., 2011[55]).

In conclusion, the effects of stress on the GI system can be classified into six different actions: GI tract movement disorders, increased visceral irritability, altered rate and extent of various GI secretions, modified permeability of the intestinal barrier, negative effects on blood flow to the GI tract, and increased intestinal bacteria counts (Konturek et al., 2011[55]).

## Stress and the Endocrine System

There is a broad and mutual relationship between stress and the endocrine system. On one hand, stress has many subtle and complex effects on the activity of the endocrine system (Sapolsky, 2002[97]; Charmandari et al., 2005[15]), while on the other hand, the endocrine system has many effects on the response to stress (Ulrich-Lai and Herman, 2009[117]; Selye, 1956[110]). Stress can either activate, or change the activity of, many endocrine processes associated with the hypothalamus, pituitary and adrenal glands, the adrenergic system, gonads, thyroid, and the pancreas (Tilbrook et al., 2000[116]; Brown-Grant et al., 1954[11]; Thierry et al., 1968[115]; Lupien and McEwen, 1997[64]). In fact, it has been suggested that it is impossible to separate the response to stress from the functions of the endocrine system. This premise has been advanced due to the fact that even a minimal amount of stress can activate the hypothalamic-pituitary-adrenal axis, which itself is intricately involved with the activation of several different hormone secreting systems (Sapolsky, 2002[97]). In different locations throughout this article, we have already discussed the effects of stress on hormones and various endocrine factors and, thus, they will not be further addressed.

## Conclusion

Altogether, stress may induce both beneficial and harmful effects. The beneficial effects of stress involve preserving homeostasis of cells/species, which leads to continued survival. However, in many cases, the harmful effects of stress may receive more attention or recognition by an individual due to their role in various pathological conditions and diseases. As has been discussed in this review, various factors, for example, hormones, neuroendocrine mediators, peptides, and neurotransmitters are involved in the body's response to stress. Many disorders originate from stress, especially if the stress is severe and prolonged. The medical community needs to have a greater appreciation for the significant role that stress may play in various diseases and then treat the patient accordingly using both pharmacological (medications and/or nutraceuticals) and non-pharmacological (change in lifestyle, daily exercise, healthy nutrition, and stress reduction programs) therapeutic interventions. Important for the physician providing treatment for stress is the fact that all individuals vary in their response to stress, so a particular treatment strategy or intervention appropriate for one patient may not be suitable or optimal for a different patient.

## Notes

Yunes Panahi and Amirhossein Sahebkar (Department of Medical Biotechnology, School of Medicine, Mashhad University of Medical Sciences, Mashhad, Iran, P.O. Box: 91779-48564, Iran; Tel: 985118002288, Fax: 985118002287, E-mail: sahebkara@mums.ac.ir, amir_saheb2000@yahoo.com) contributed equally as corresponding authors.

## Conflict of interest

The authors declare that have no conflict of interest in this study.

## Acknowledgement

The authors would like to thank the "Neurosciences Research Center of Baqiyatallah University of Medical Sciences" and the “Clinical Research Development Center of Baqiyatallah (a.s.) Hospital” for providing technical supports.

## Figures and Tables

**Table 1:**
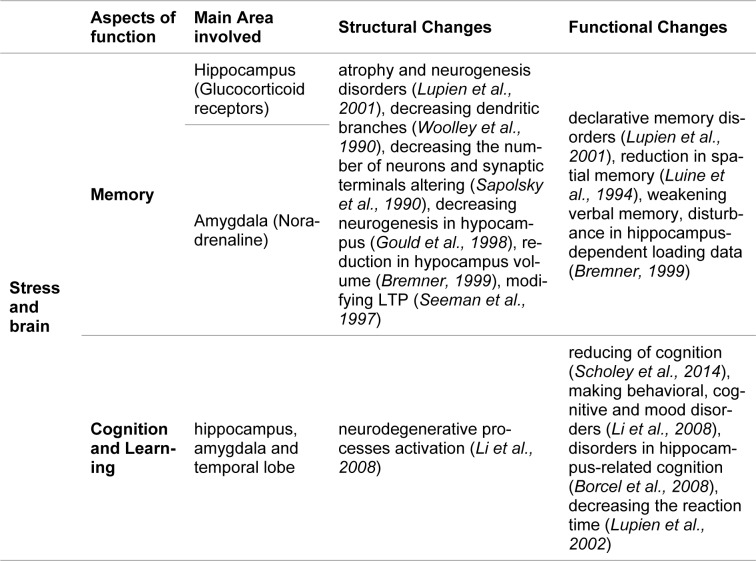
Destructive effects of stress of CNS function

**Table 2:**
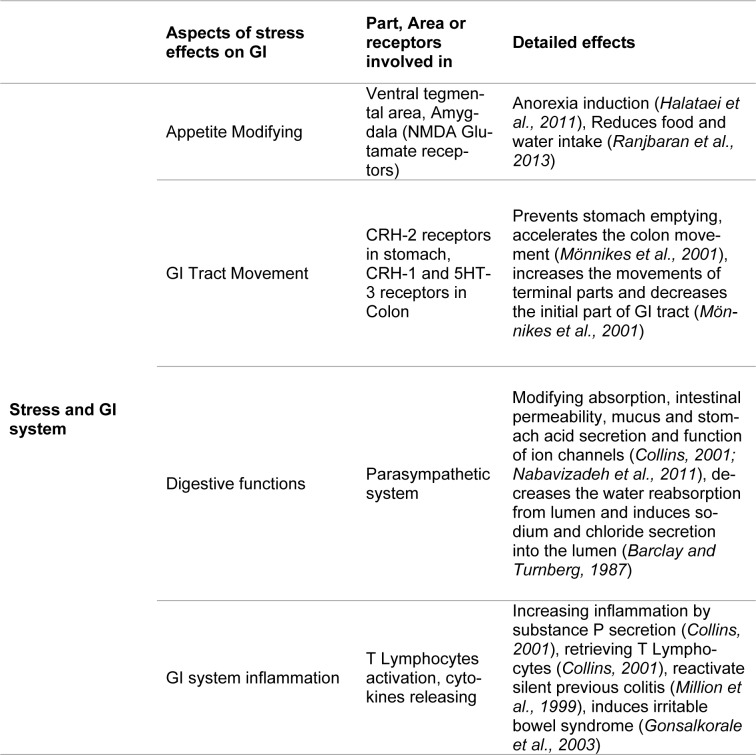
Stress has various effects on the function of GI system
